# “Heart Oddity”: Intrinsically Reduced Excitability in the Right Ventricle Requires Compensation by Regionally Specific Stress Kinase Function

**DOI:** 10.3389/fphys.2020.00086

**Published:** 2020-02-18

**Authors:** Alexey V. Zaitsev, Mark Warren

**Affiliations:** Nora Eccles Harrison Cardiovascular Research and Training Institute, University of Utah, Salt Lake City, UT, United States

**Keywords:** right ventricle, right ventricle outflow tract, PKA, CaMKII, phosphatase, conduction velocity, ventricular fibrillation, Brugada syndrome

## Abstract

The traditional view of ventricular excitation and conduction is an all-or-nothing response mediated by a regenerative activation of the inward sodium channel, which gives rise to an essentially *constant* conduction velocity (CV). However, whereas there is no obvious biological need to tune-up ventricular conduction, the principal molecular components determining CV, such as sodium channels, inward-rectifier potassium channels, and gap junctional channels, are known targets of the “stress” protein kinases PKA and calcium/calmodulin dependent protein kinase II (CaMKII), and are thus *regulatable* by signal pathways converging on these kinases. In this mini-review we will expose deficiencies and controversies in our current understanding of how ventricular conduction is regulated by stress kinases, with a special focus on the chamber-specific dimension in this regulation. In particular, we will highlight an odd property of cardiac physiology: uniform CV in ventricles requires co-existence of mutually opposing gradients in cardiac excitability and stress kinase function. While the biological advantage of this peculiar feature remains obscure, it is important to recognize the clinical implications of this phenomenon pertinent to inherited or acquired conduction diseases and therapeutic interventions modulating activity of PKA or CaMKII.

## Introduction

Traditionally, conduction through the ventricular tissue has been considered an all-or-none event scantly amenable to control. However, as early as 1953 Siebens et al. (1953) showed that sympathetic agonists modestly accelerated ventricle conduction. Using diverse approaches, subsequent studies also showed modest conduction increases in response to adrenergic stimulation (Munger et al., 1994; de Boer et al., 2007; Lang et al., 2015; Ajijola et al., 2017). Of interest, the duration of the QRS complex, which reflects the total time of conduction spread through the ventricles (Nattel and Jing, 1989), may change dynamically in the 24 h cycle (Nakagawa et al., 1998), or in response to exercise (Pilhall et al., 1992). Other studies suggest that sex hormones modulate QRS duration (Macfarlane et al., 1994; Okin et al., 1995). Interestingly, the conductivity of channels involved in the generation and transmission of the ventricular impulse (notably the cardiac Na^+^ channel, Nav1.5) may be modulated by phosphorylation, and thus are amenable to regulation by protein kinases responding to various neural and hormonal signals, in particular transmitted through activation of G-protein-coupled receptors (GPCRs) (Sato et al., 2015). Prominent in these signaling pathways are the calcium/calmodulin-dependent protein kinase II (CaMKII) (Hund et al., 2010; Ashpole et al., 2012; Glynn et al., 2015; Burel et al., 2017) and the cAMP-activated protein kinase A (PKA) (Murphy et al., 1996; Zhou et al., 2002; Aiba et al., 2014), collectively known as “stress” kinases for their involvement in the “fight or flight” physiological response (Wehrens et al., 2004; Wu et al., 2016). This mini-review will focus on very recent (and still limited) information regarding how the electrical wave propagation through ventricular chambers is regulated by stress kinases. Specifically, we will highlight a largely unknown *regional* aspect of kinase function in the ventricles, and will discuss its relevance to clinical conditions causing reduced ventricular excitability, such as the Brugada syndrome (BrS). A comprehensive review on the fundamentals of cardiac conduction can be found elsewhere (Veeraraghavan et al., 2014).

## Normal Ventricular Conduction Is Sustained by Constitutive Activity of CaMKII and PKA

CaMKII, a serine/threonine-specific protein kinase regulated by the Ca^2+^/calmodulin complex (Maier and Bers, 2002; Erickson, 2014), modulates the cardiac response to stress by targeting numerous ion channels and transporters (Bers and Grandi, 2009). Importantly, CaMKII functionally regulates the three main components of cardiac excitability: Nav1.5 (Wagner et al., 2006; Yoon et al., 2009; Aiba et al., 2010), inward-rectifier potassium channels underlying the K^+^ current I_K__1_ (Wagner et al., 2009), and gap junction channels formed by Connexin 43 (Cx43) proteins (Procida et al., 2009; Huang et al., 2011). Recently, CaMKII has garnered attention due to its ability to modulate ion channels in ways that favor afterdepolarizations, and for its prominent role in cardiac disease development (Swaminathan et al., 2012). Increased activity of CaMKII [which may occur due to CaMKII overexpression (Zhang et al., 2002, 2003) or upregulation in the failing heart (Anderson, 2005)] alters Ca^2+^ homeostasis [including increased Ca^2+^ entry through I_CaL_ (Anderson et al., 1994); increased Ca^2+^ release through RyR (Wehrens et al., 2004); and increased Ca^2+^ reuptake to the SR (Mattiazzi and Kranias, 2014)] and enhances the late sodium current (I_NaL_) (Wagner et al., 2006; Maltsev et al., 2008), both effects promoting abnormal cellular triggered activity and arrhythmia (Anderson, 2005; Vincent et al., 2014). Whereas there is a general consensus on the direction of CaMKII regulation of cellular Ca^2+^ cycling (Swaminathan et al., 2012), regulation of cellular excitability and conduction by CaMKII remains controversial. On the cellular level, some studies suggested that CaMKII activity favors an overall Na^+^ current (I_Na_) upregulation (Yoon et al., 2009; Aiba et al., 2010), while others argued that it promotes an overall I_Na_ downregulation (Wagner et al., 2006). Yoon et al. (2009) were the first to suggest that baseline CaMKII activity is *required* for normal ventricular excitation, and that CaMKII inhibition is detrimental. The authors showed that the CaMKII blocker KN93 reduced peak I_Na_, shifted the steady-state inactivation curve to hyperpolarized values, decreased I_*NaL*_, enhanced intermediate inactivation, and delayed the recovery from fast and slow inactivation. Altered I_Na_ kinetics led to a significant suppression of the action potential upstroke velocity (d*V*/d*t*_*max*_), a measure of cellular excitability. In terms of the regulation *direction*, Yoon et al.’s (2009) results are consistent with findings by Aiba et al. (2010), who showed that intracellular delivery of CaMKII (CaMKIIα) to isolated guinea pig myocytes caused upregulation of I_Na_ (i.e., changes in kinetics leading to increased availability of I_Na_ under physiological conditions). However, the studies by Yoon et al. (2009) and Aiba et al. (2010) contradict the studies performed in isolated adult mouse/rabbit myocytes (Wagner et al., 2006), HEK293 cells (Deschenes et al., 2002; Ashpole et al., 2012), or using simulations (Hund et al., 2008), that showed that overexpression/inhibition of CaMKII suppressed/enhanced I_Na_ availability, and inhibitors KN93 (or AIP, a peptide inhibitor) rescued CaMKII overexpression-induced detrimental effects.

**FIGURE 1:**
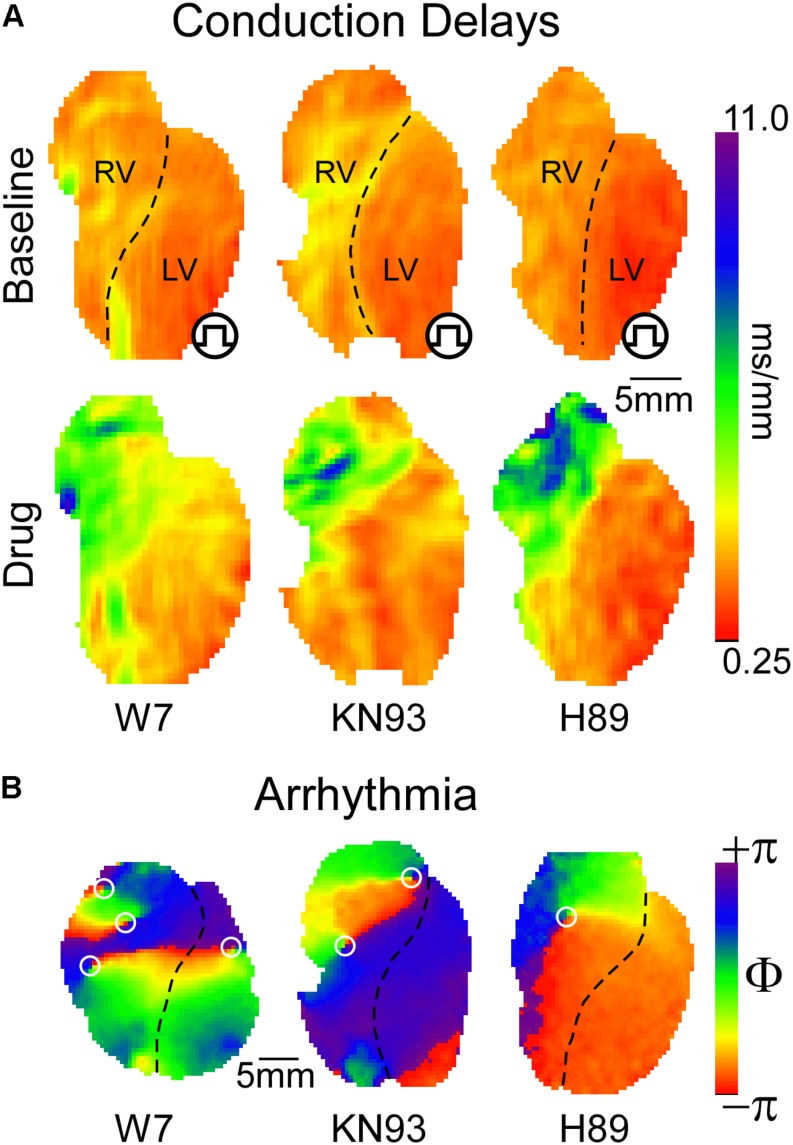
**(A)** Inhibition of either CaMKII or PKA signaling leads to preferential conduction slowing in the basal RV approximately corresponding to the RVOT. Qualitatively, similar effects are produced by blockade of calmodulin, CaMKII, or PKA with W7, KN93, and H89, respectively. **(B)** With sufficient concentration and/or time of exposure, all three interventions lead to reentrant VT or VF, with the driving rotors preferentially occurring in the RVOT [reused with permission from the American Journal of Physiology (Warren and Zaitsev, 2017) and Zaitsev et al. (2019)].

CaMKII regulation of ventricular conduction in the whole heart is also controversial. Takanari et al. (2016) reported that a chronic reduction in CaMKII activity in mice following expression of CaMKII-inhibiting autocamtide-3-related peptide (AC3-I) caused an *increase* in conduction velocity (CV) in both RV and LV. In addition, they showed that inhibition of calmodulin, the upstream regulator of CaMKII, *increased* ventricular CV, and *reduced* arrhythmogenicity in isolated rabbit hearts (Takanari et al., 2016). The improved conduction following calmodulin/CaMKII inhibition was attributed to increased localization of Cx43 in the intercalated disk (Takanari et al., 2016). On the physiological level, our own studies yielded strikingly opposite outcomes (Warren and Zaitsev, 2017; Warren et al., 2017; Zaitsev et al., 2019). Specifically, inhibition of either CaMKII or calmodulin *slowed down* propagation mainly due to inducing severe conduction defects in the right ventricular outflow tract (RVOT, Figure 1A, leftmost and center panels), and this was *proarrhythmic* (Figure 1B; Warren and Zaitsev, 2017; Zaitsev et al., 2019). Whilst Cx43 channel function was not analyzed, CaMKII blockade reduced d*V*/d*t*_max_ both in myocytes and whole hearts (Warren et al., 2017; Zaitsev et al., 2019), consistent with Yoon et al.’s (2009) data, and suggestive of a reduced I_Na_ availability. Moreover, CaMKII inhibition caused highly rate-dependent changes of ventricular conduction and excitability (see more below) (Warren et al., 2017; Zaitsev et al., 2019), readily explained by altered I_Na_ inactivation (Yoon et al., 2009), but not by altered localization or conductivity of Cx43. Investigating how CaMKII gain- and loss-of-function alters both myocardial active and passive properties in the same whole-heart animal model will likely resolve the controversy. We invite anyone interested to collaborate on such a study.

PKA is a cAMP sensitive protein kinase which responds to beta-adrenergic receptor activation (Taylor et al., 2013; Soni et al., 2014), and is inhibited by muscarinic receptor activation (Harvey and Belevych, 2003). PKA activation drives the physiological response to stress by targeting molecular components of cardiac function and excitability (Soni et al., 2014). Interestingly, CaMKII and PKA work hand in hand *upregulating* excitation–contraction coupling in response to stress (Grimm and Brown, 2010). Consequently, and given that Nav1.5 phosphorylation sites targeted by CaMKII or PKA cluster mostly in the first intracellular linker loop of the channel (Marionneau and Abriel, 2015), including a potentially shared site at Ser^571^ (Hund et al., 2010; Marionneau et al., 2012), we hypothesized that PKA regulates ventricular conduction similarly to CaMKII. This was subsequently confirmed (Zaitsev et al., 2019). Figure 1A (rightmost panel) illustrates how PKA blockade with H89 caused a highly non-uniform, RVOT-centric, depression of conduction, which initiated arrhythmia (Figure 1B).

Our organ-level findings are generally consistent with single cell patch clamp studies indicating that PKA and/or its upstream signals (beta-agonists and cAMP) *upregulate* I_Na_ (Matsuda et al., 1992; Frohnwieser et al., 1997; Lu et al., 1999; Aiba et al., 2010). Although some early studies suggested opposite effects (Ono et al., 1989; Schubert et al., 1989), slow background shifts in I_Na_ kinetics common in isolated myocytes may have confounded those results (Ono et al., 1993). Of note, diverse mechanisms of PKA-mediated I_Na_ upregulation were proposed, including PKA-induced increase in Na^+^ channel trafficking to the sarcolemma (Zhou et al., 2000, 2002). To date, there is no cellular counterpart to our whole heart study (Zaitsev et al., 2019) that would clarify how *inhibition* of PKA modulates I_Na_ gating. However, similarities in the conduction depression patterns elicited by CaMKII and PKA inhibition (Figure 1) suggest that the underlying ionic mechanisms are also similar.

## Role of Phosphatases – A “Known Unknown”

In the 1980s the notion emerged that opposing actions of endogenous phosphatases and associated kinases set the basal level of membrane currents, such as the L-type inward Ca^2+^ current (Hescheler et al., 1988), I_K__1_ (Koumi et al., 1995a), and connexins (Moreno et al., 1994). It is suggested that type 1 and type 2A phosphatases (PP1 and PP2A) are key to regulating ion channel phosphorylation (Luss et al., 2000; Terentyev and Hamilton, 2016), but improved understanding is pending. A recent study by El Refaey et al. (2019) using a PP2A phosphatase-defective transgenic mouse showed that adrenergic stimulation of myocytes induced aberrant action potentials attributed to a deficient dephosphorylation of Nav1.5 affecting I_*NaL*_. This study reported no significant difference in the fast component of I_Na_ (El Refaey et al., 2019).

In our experiments, administration of broad-acting phosphatase inhibitor calyculin to isolated rabbit hearts led to a small yet significant acceleration of ventricular conduction, the effect being greater in the RV than in the LV (Zaitsev et al., 2019). This supports a phosphatase-mediated *negative* regulation of excitability, which differs from El Refaey et al. (2019). Given the broad action of calyculin, our data might indicate that a phosphatase other than PP2A (e.g., PP1) regulates the fast component of I_Na_.

Additional indirect evidence of the phosphatase-mediated *negative* regulation of excitability is the progressive nature of CaMKII/PKA-inhibition-mediated conduction defects (steady state after >1 h of kinase blockade) (Zaitsev et al., 2019). The progressive conduction deterioration is most easily explained in terms of continuing phosphatase activity amid decreased total kinase activity. Because the RV is revealed as the most vulnerable region, it is plausible that phosphorylation/dephosphorylation imbalance affects it more prominently.

## Stress Kinases Regulate Ventricular Conduction in a Rate-Dependent Manner

Besides the progressive conduction deterioration caused by CaMKII and/or PKA blockade, the effect of each blocker was strongly rate dependent. Combined, these two effects resulted in a frequency-dependence which progressed with time. At short durations of drug exposure, the detrimental effect of either CaMKII or PKA blockade was noticeable only at relatively high pacing rates (short pacing intervals), but with increased exposure to drug conduction was affected even at physiological pacing rates. The detrimental effects of kinases’ blockade were always largest in the basal RV (approximately corresponding to the RVOT), culminating in 2:1 conduction block, turbulence, and initiation of VF in that region at pacing intervals as long as 400 ms (Figure 1B; Zaitsev et al., 2019). Remarkably, severe conduction depression induced by either CaMKII or PKA inhibition was almost fully abolished just by prolongation of the pacing interval to >6000 ms (Zaitsev et al., 2019).

This result is revealing. First, it suggests that I_K__1_ or Cx43 channels, the two major factors of ventricular syncytial conduction besides I_Na_, do not play a significant role in mediating adverse effects caused by stress kinases’ inhibition. Even though current evidence points to CaMKII and PKA targeting both connexins (Burt and Spray, 1988; Moreno et al., 1992; Procida et al., 2009; Huang et al., 2011) and I_K__1_ (Fakler et al., 1994; Koumi et al., 1995a,b,c; Wagner et al., 2009), neither is known to possess time-dependent gating properties. Second, given Yoon et al.’s (2009) data, enhancement of intermediate/slow inactivation of I_Na_ emerges as the likely mechanism causing conduction abnormalities in the presence of CaMKII inhibition. Furthermore, by similarity of the CaMKII and PKA inhibition effects, we predict that PKA also regulates intermediate/slow inactivation of I_Na_, an effect not reported thus far. Lastly, the fact that long period of quiescence abolishes the effect of PKA and/or CaMKII inhibition disfavors the notion that our observed upregulation of I_Na_ by PKA and/or CaMKII is due to Na^+^ channel trafficking to the plasma membrane (Zhou et al., 2000). From the perspective of Nav1.5 gating mechanisms, it remains to be understood how insufficient phosphorylation of the protein, likely at locations identified in the 1st intracellular loop (Marionneau and Abriel, 2015), favors stabilization of the channel in the inactivated state.

## Constitutive Activity of CaMKII and PKA in the Heart – How Much of It?

De Koninck and Schulman (1998) demonstrated that CaMKII can act as an intracellular Ca^2+^ ([Ca^2+^]_*i*_) transducer and activation frequency sensor *in vitro*. In line with this, using FRET-based biosensor Camui, Erickson et al. (2011) showed that increased activation rates (range 0–1 Hz) significantly increased CaMKII activity in isolated rabbit ventricular myocytes. Since the tested rates are much below the resting heart rate, one should expect a much higher level of CaMKII activation at the physiological baseline. In addition, even in quiescent cells, a significant activation of CaMKII produced by various neurohumoral ligands acting upon GPCR was reported (Erickson et al., 2011). Thus, the normal physiological level of CaMKII activity in the heart is a complex integral of various signals. Whereas it has not yet been quantitatively assessed, the degree at which CaMKII blockade affects conduction in hearts paced at normal physiological rate and in the absence of GPCR stimulation (Zaitsev et al., 2019), clearly indicates that the basal level of CaMKII activity is far from zero. To which extent this level is regulated by acute variations in heart rate and [Ca^2+^]*_*i*_*, or by phasic changes in autonomic and endocrine regulation, remains unknown and needs to be further investigated.

PKA constitutive activity is largely determined by the relationship between the half-maximal cAMP concentration required for PKA activation and the basal level of cAMP in cells. Measures of cAMP concentration yielded values around 1 μM (Terasaki and Brooker, 1977; Iancu et al., 2008). According to several *in vitro* studies, the cAMP concentration required for half-maximal activation of PKA is in the range 90–300 nM (Adams et al., 1991; Mongillo et al., 2004). Based on these estimates, PKA should be fully activated under resting conditions, precluding the possibility of a dynamic response to upstream signals acting via cAMP, which clearly contradicts fundamental physiology. Various schemes were proposed to resolve this controversy, including cAMP sequestration that renders it inactive (Exton et al., 1971), or the presence of intracellular domains in which cAMP concentration is much lower than the bulk cytosol concentration (Iancu et al., 2008). Recently, Koschinski and Zaccolo (2017) challenged the prior *in vitro* estimates of PKA sensitivity, reporting significantly higher half-maximal concentrations for the enzyme (5.2 μM cAMP) measured in intact Chinese hamster ovary cells. Upon inhibition of PKA (10 μM H89) in unstimulated cells, they found no detectable change in FRET-based PKA activity reporter signal, suggesting a negligible level of PKA activity at baseline cAMP levels. However, 10 μM H89 induced a dramatic slowing of ventricular conduction in perfused rabbit hearts, supporting the presence of robust endogenous PKA activity, even in the absence of beta-adrenergic and other neural and hormonal signals (Zaitsev et al., 2019). Perhaps, PKA signaling is very different between ovary cells and cardiac myocytes. Since isolated hearts respond to both beta-adrenergic stimulation and stimulation of cAMP synthesis with vigorous increase in PKA activity (Zhang and MacLeod, 1996), the constitutive level of PKA activity in the intact heart must be far from both inactive and fully activated state, but where it stands exactly, remains to be established.

## Conduction Vulnerability in the RV: The Achilles’ Heel of the Heart

The spatial patterns of ventricular conduction depression induced by “stress” kinase inhibitors are unique insomuch that the RV is affected much more prominently than the LV, and within the RV the most affected region is RVOT. This pattern is remarkably similar among the kinase inhibitors KN93 and H89, as well as the calmodulin inhibitor W7 (Figure 1A), suggesting a common mechanism of action.

**FIGURE 2:**
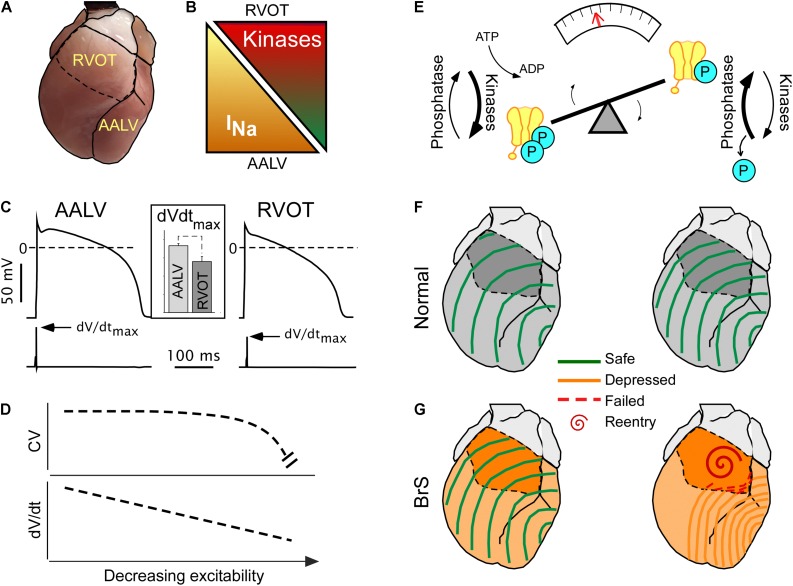
Concealed conduction vulnerability in the right ventricular outflow tract (RVOT). The left-hand part **(A–D)** illustrates the currently established static component of the phenomenon, whereas the right-hand part **(E–G)** illustrates a largely hypothetical dynamic component. **(A)** The anterior view of the heart showing RVOT and the anterior–apical left ventricle (AALV), the two regions with demonstrated significant differences in properties affecting conduction. **(B)** Compared to AALV (and possibly other ventricular regions), RVOT has a lesser expression of Nav 1.5, the pore-forming subunit of I_Na_ (Boukens et al., 2013), but a larger expression of “stress” kinases PKA and CaMKII (Zaitsev et al., 2019). **(C)** Cardiac myocytes from RVOT have reduced excitability (assessed by the maximum slope of the action potential upstroke slope, d*V*/d*t*_max_) than cardiac myocytes from AALV [reused with permission from the American Journal of Physiology (Warren et al., 2017)]. **(D)** The theoretical non-linear relationship between excitability and conduction velocity (Shaw and Rudy, 1997) which may explain the relatively uniform conduction velocities across the ventricles (Zaitsev et al., 2019) despite the regional differences in the expression of Nav 1.5 and excitability [modified Shaw and Rudy (1997), with permission]. **(E)** A diagram highlighting the dynamic nature of the phosphorylated state of Nav 1.5, which depends on the dynamic equilibrium between the sum of all kinase and phosphatase activity. Overall, little is known about the actual temporal fluctuations of Nav 1.5 phosphorylation, but such can be inferred from the fact that both PKA and CaMKII are downstream of highly dynamic autonomic and endocrine signals. **(F,G)** Physiological fluctuations of Nav 1.5 phosphorylation may lead to variations in excitability and conduction velocity [slower conduction with less phosphorylation (Zaitsev et al., 2019)]. While such variations are benign under normal conditions **(F)**, they may lead to acute conduction crisis and reentrant arrhythmias when I_Na_ expression is globally reduced **(G)** (Remme and Wilde, 2014), or when the phosphorylation balance of Nav 1.5 is compromised (Aiba et al., 2014) in patients with Brugada syndrome (BrS).

Figures 2A–C illustrate how the specific RVOT vulnerability can be explained in terms of its intrinsically reduced excitability (reflected in reduced d*V*/d*t*_max_, see Figure 2C inset), compared to other ventricular regions such as the anterior–apical left ventricle (AALV) (Warren et al., 2017). The reduced excitability presumably results from the locally reduced expression levels of Nav1.5 (Veeraraghavan and Poelzing, 2008; Boukens et al., 2013). The fact that, despite regional differences in excitability, the impulse conduction is uniform through the ventricular chambers (see Figure 1A, upper panels; see also Figures 3D–E in Boukens et al., 2013) requires explanation. We believe that two factors are involved. One is that cardiac excitability operates within a wide margin of safety across which impulse conduction is robust (Figure 2D). The presence of such a safety margin stems from the asymptotic relationship between action potential upstroke (d*V*/d*t*_max_) and conduction (Buchanan et al., 1985; Shaw and Rudy, 1997), whereby d*V*/d*t*_max_ needs to cross a critical threshold before conduction is noticeably affected (Figure 2D).

The second factor is the presence of spatial heterogeneity in stress kinase-related signaling (Figures 2A,B). We found that the protein expression levels of CaMKII-δ (Maier and Bers, 2002) and of the catalytic subunit of PKA (PKA-Cα) (Yin et al., 2008) are significantly higher in the RVOT than in the AALV (Zaitsev et al., 2019), suggesting increased local activities of these enzymes. Others showed that the RV hemodynamic response was more sensitive to β-adrenergic stimulation than the LV (Irlbeck et al., 1996). Additionally, RV myocytes subject to isoproterenol exhibited increased sarcomere shortening, Ca^2+^ transient amplitude, cytoplasmic cAMP accumulation, and PKA activity compared to the LV counterparts (Molina et al., 2014). These findings suggest the existence of an organ-wide program which controls local cellular signaling to maintain a specific functional profile. Whereas the physiological advantage of such an adaptation remains unclear, an apparent physiological role of stress kinases is to upregulate functional I_Na_ (Aiba et al., 2010) to maintain RV excitability well within the margins of safety. The source of this heterogeneity may be linked to the distinct development origin of the RVOT (Boukens et al., 2009), which has been associated to a more persistent RVOT-regional slow impulse conduction during cardiac development (de Jong et al., 1992; Boukens et al., 2013). Alternatively, we speculate that higher expression levels of CaMKII and PKA in RVOT could develop in response to a comparatively higher local mechanical stress caused by the distinct hemodynamic context in which RVOT operates (Geva et al., 1998). However, the physiological advantage or necessity of intrinsically reduced RVOT excitability (Warren et al., 2017) remains elusive.

Finally, the role of regional differences in the myocardial organization properties such as connexin distribution (Ou et al., 2005), fiber orientation (Burgess et al., 1988), or cleft geometry (Kelly et al., 2018) might be important. We should note, however, that isolated cells subject to kinase inhibitors developed depressed excitability characteristics akin to that observed in intact tissue (Warren et al., 2017), indicating that tissue architecture *is not required* for the phenomenon to develop. Additionally, the dynamic nature of the phenomenon cannot be readily ascribed to tissue architecture components which are functionally time-independent. Thus, it seems that the tissue architecture is not directly responsible for the core phenomenon of kinase inhibition-induced conduction depression. However, it may contribute to increased conduction vulnerability in the RVOT by conferring the region a narrower safety margin for conduction.

## Stress Kinase Activity and the Brugada Syndrome

The BrS is a hereditary lethal cardiac condition associated with conduction abnormalities in the RVOT (Kasanuki et al., 1997; Postema et al., 2010; Zhang et al., 2015). Abnormal I_Na_ function likely underlies the BrS phenotype, since the majority of known mutations causing the disease affect this current (Antzelevitch et al., 2017). The ECG signature of BrS, an abnormal ST segment elevation in precordial leads, manifests intermittently (Veltmann et al., 2006). Circadian biases in the development of abnormal ST segment elevation (Gray et al., 2017), as well as in the initiation of VF (Matsuo et al., 1999), underscore the dynamic nature of BrS. Increased parasympathetic tone can also underlie the dynamic unmasking of the disease phenotype, which can be reverted by interventions that increase the sympathetic signals (Kasanuki et al., 1997).

Conduction patterns induced by “stress” kinase inhibition (Figure 1) are strikingly similar to RVOT-centric conduction defects described in BrS patients (Zhang et al., 2015). A prevailing paradox is that the permanent nature of dysfunctional Nav1.5 mutations afflicting BrS patients is typically associated to intermittent or lacking phenotypes. We speculate that the intermittent display of BrS phenotype may in part depend on dynamic fluctuations in stress kinases’ activity. Supporting this, half-maximal blockade of I_Na_ with TTX [which is similar to the degree of I_Na_ loss-of-function in some BrS models (Papadatos et al., 2002; Remme et al., 2009)] caused only a uniform slowing of conduction, while a subsequent short exposure to CaMKII inhibitor KN93 disproportionally slowed RV conduction, bringing about BrS-like phenotype (Zaitsev et al., 2019). We submit that following kinase activity fluctuations (Figure 2E), ventricular excitability is dynamically shifted back-and-forth within the limits of safe conduction, which are wide in healthy hearts (Figure 2F), but narrower in hearts affected by the BrS (Figure 2G). When this limit is breached, conduction in the RVOT fails first, and VF initiates in a manner similar to that observed during kinase inhibition (Figure 2G, right panel). Whereas there are no known cases of BrS involving CaMKII signaling, a family with BrS bore a SCN5A mutation in a PKA consensus phosphorylation site, which effectively disrupted positive regulation of I_Na_ by PKA (Aiba et al., 2014). Overall, the role of stress kinase signaling in BrS remains poorly understood and definitely merits further investigation.

## Conclusion

Because of an intrinsically reduced safety margin for conduction in the RV/RVOT, the constitutive activity of both CaMKII and PKA is *required* for normal ventricular conduction. Consequently, any intervention decreasing activity of these kinases is potentially pro-arrhythmic and life-threatening. Further, any condition leading to additional reduction in the RV excitability (BrS, ischemia, and electrolyte imbalance) bears increased risk. Normal ventricular conduction hinges on the delicate balance of phosphorylation/dephosphorylation, which is a result of a very complex and highly dynamic summation of upstream signals mediated through nervous and endocrine regulation, as well as circadian rhythms. Whereas a wealth of knowledge has been accumulated at the level of molecular mechanisms involved in regulation of cardiac ionic channels by phosphorylation, there is a deficiency in translating these mechanisms to the level of whole-heart physiology and pathophysiology. We hope that this mini-review will stimulate investigations to bridge this gap.

## Author Contributions

Both authors contributed equally to the preparation of this review.

## Conflict of Interest

The authors declare that the research was conducted in the absence of any commercial or financial relationships that could be construed as a potential conflict of interest.
